# Commentary on: Peng, Y et al., Structured recurrent inhibition in the presubiculum could improve information processing

**DOI:** 10.1007/s00424-021-02626-y

**Published:** 2021-10-14

**Authors:** Gilda Baccini, Peer Wulff

**Affiliations:** Institute of Physiology, Christian-Albrechts-University Kiel, 24098 Kiel, Germany

Neuronal connectivity provides the basis for brain function. Cortical microcircuits consist mainly of excitatory output neurons or principal cells and a smaller number of diverse inhibitory interneurons. Whereas principal cells (PCs) are generally assumed to sparsely and preferentially innervate their target neurons, the organization of interneuron connectivity is still a matter of debate. A recent paper of Peng and colleagues, published in Science Advances, makes a very stimulating contribution to this discussion [9].

A prevailing view suggests that inhibitory interneurons cover neighboring principal cells (PCs) evenly in a “blanket of inhibition.” In this model, interneurons, belonging to different subtypes, innervate neighboring PCs densely and non-selectively [5, 8]. Indeed, this configuration can support a variety of functions attributed to interneurons, such as input discrimination through lateral inhibition, dynamic range extension through feedforward inhibition, or the generation of oscillations [7, 11]. However, in some cortical regions, interneuron subtypes were reported to show selective connections to PCs, forming subnetworks. Basket cells in the hippocampal CA1 region and in layer II of the medial entorhinal cortex, for example, show selective connectivity with neighboring PCs based on the principal cells’ projection targets [1, 12], which may aid the specific routing of information. In the dentate gyrus, on the other hand, parvalbumin-positive interneurons preferentially mediate lateral inhibition of PCs, rather than recurrent (feedback) inhibition, which may aid the differentiation between similar inputs [4].

**Fig. 1:**
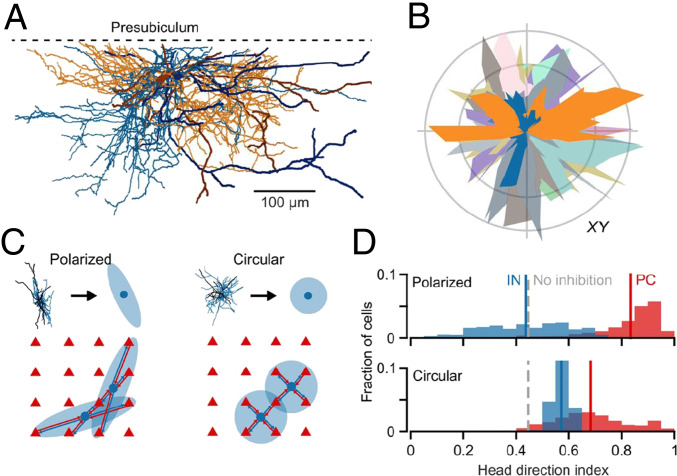
Polarized axons of PV interneurons may promote head direction tuning of principal cells in the presubiculum. **A** 3D reconstructions of two different parvalbumin-positive interneurons in the superficial presubiculum illustrating the polarity of their axons (orange and blue). **B** Polar plots of axonal distributions for the two interneurons in **A** (orange and blue) and for an additional 10 parvalbumin-positive interneurons show that axons are oriented in all directions. **C** Cartoon illustrating the layout of network models with either polarized or circular connectivity. In both models, PCs were connected reciprocally with interneurons along the extent of the interneurons’ axonal clouds. Polarized interneuron axons were oriented to increase the diversity of head direction preferences of connected PCs. **D** Head direction indices of PCs (red) and interneurons (blue) show that polarized interneuron axons improve head direction tuning of PCs and broaden head direction tuning of interneurons compared to circular axons, which represent a blanket of inhibition model. The figure is adapted from Figs. 3 and 4 of reference [9]

Peng and colleagues give yet another impressive example of such non-random inhibition and at the same time offer an intriguing functional implication such “structured” inhibition may have [9]. Using multi-patch recordings in the rat superficial presubiculum, the authors initially found that only a minority of interneurons (parvalbumin-positive and -negative subtypes) were highly interconnected with PCs, whereas others had only few or no connections with PCs. In addition, recurrent (reciprocal) connections between highly connected interneurons and PCs were more frequent than would be expected for random connectivity. Subsequent 3D reconstructions of recorded cell clusters revealed that the axonal arbor of interneurons was asymmetrically arranged in ellipsoids rather than spheres (Fig. 1 A and B), resulting in the selective inhibition of PCs within the arborization volume. These polarized axon clouds differed markedly from the symmetrical axonal arborizations of parvalbumin-expressing interneurons in the entorhinal cortex, which show random inhibitory connectivity [3]. Very notably, in the presubiculum, the same PCs that received inhibitory input from a specific interneuron also excited this particular interneuron in a spatially directed reciprocal connectivity motif. In addition, the long axis of the polarized axonal clouds of individual interneurons varied to cover all possible orientations in space, which explained the abundance of interneurons without apparent connections in the single geometrical plane of the multi-patch recording approach.

To explore potential functional implications of such polarized connectivity between interneurons and principal cells, the authors designed a presubicular network model (Fig. 1 C) for the processing of head direction information—a major function of this region [6, 13]. Interneurons and PCs were evenly spaced on a 2D grid and topographical head direction inputs were assumed so that the position of a neuron determined its head direction preference. PCs and interneurons were reciprocally connected following the observed rules of structured inhibition. An additional assumption was that the polarized axons of individual interneurons were oriented such that they connected to PCs with maximally diverse head direction preferences. For comparison, the authors generated a blanket of inhibition model based on random connectivity, where axon arborization was symmetrical and interneuron sampling was constrained to neighboring PCs (Fig. 1 C). Interestingly, simulations showed that polarized inhibition improved the head direction tuning of PCs. At the same time, directional tuning of the interneurons themselves broadened (Fig. 1 D), in line with previous reports from cortical regions [2, 10]. These effects on head direction tuning are likely explained by reciprocal connectivity of interneurons with PCs of different input preferences.

In summary, Peng and colleagues have provided a striking example of structured inhibition and offer an inspiring functional implication such spatially asymmetric connectivity may have to realize specific computations at the level of the microcircuit.
